# JAK/STAT3 signaling in cardiac fibrosis: a promising therapeutic target

**DOI:** 10.3389/fphar.2024.1336102

**Published:** 2024-03-01

**Authors:** Heng Jiang, Junjie Yang, Tao Li, Xinyu Wang, Zhongcai Fan, Qiang Ye, Yanfei Du

**Affiliations:** ^1^ Department of Cardiology, The Affiliated Hospital of Southwest Medical University, Luzhou, Sichuan, China; ^2^ Key Laboratory of Medical Electrophysiology, Ministry of Education and Medical Electrophysiological Key Laboratory of Sichuan Province, Institute of Cardiovascular Research, Southwest Medical University, Luzhou, China

**Keywords:** cardiovascular diseases, JAK/STAT3 signaling, cardiac fibrosis, cardiac fibroblast proliferation and activation, signal transduction and regulation, upstream mediators, anti-fibrotic therapies

## Abstract

Cardiac fibrosis is a serious health problem because it is a common pathological change in almost all forms of cardiovascular diseases. Cardiac fibrosis is characterized by the transdifferentiation of cardiac fibroblasts (CFs) into cardiac myofibroblasts and the excessive deposition of extracellular matrix (ECM) components produced by activated myofibroblasts, which leads to fibrotic scar formation and subsequent cardiac dysfunction. However, there are currently few effective therapeutic strategies protecting against fibrogenesis. This lack is largely because the molecular mechanisms of cardiac fibrosis remain unclear despite extensive research. The Janus kinase/signal transducer and activator of transcription (JAK/STAT) signaling cascade is an extensively present intracellular signal transduction pathway and can regulate a wide range of biological processes, including cell proliferation, migration, differentiation, apoptosis, and immune response. Various upstream mediators such as cytokines, growth factors and hormones can initiate signal transmission via this pathway and play corresponding regulatory roles. STAT3 is a crucial player of the JAK/STAT pathway and its activation is related to inflammation, malignant tumors and autoimmune illnesses. Recently, the JAK/STAT3 signaling has been in the spotlight for its role in the occurrence and development of cardiac fibrosis and its activation can promote the proliferation and activation of CFs and the production of ECM proteins, thus leading to cardiac fibrosis. In this manuscript, we discuss the structure, transactivation and regulation of the JAK/STAT3 signaling pathway and review recent progress on the role of this pathway in cardiac fibrosis. Moreover, we summarize the current challenges and opportunities of targeting the JAK/STAT3 signaling for the treatment of fibrosis. In summary, the information presented in this article is critical for comprehending the role of the JAK/STAT3 pathway in cardiac fibrosis, and will also contribute to future research aimed at the development of effective anti-fibrotic therapeutic strategies targeting the JAK/STAT3 signaling.

## 1 Introduction

Cardiovascular disease is still the major cause of global death despite great progress in treatment methods. Myocardial fibrosis is a common pathology of most cardiovascular diseases at the end stage (Rockey et al., 2015). It can destroy the cardiac structure, impair cardiac excitation-contraction coupling, and impede cardiac function of both contraction and relaxation, thereby promoting the development of cardiovascular disease into heart failure (Gyöngyösi et al., 2017; Nguyen et al., 2017). The order of severity of cardiac fibrosis is related to higher long-term mortality of cardiovascular disease, particularly heart failure (Azevedo et al., 2010; Aoki et al., 2011). Due to the complex and incompletely elucidated mechanisms of fibrosis, there is currently no specific antifibrotic treatment available for cardiac fibrosis.

The Janus kinase/signal transducer and activator of transcription (JAK/STAT) signaling pathway, as a central communication node within cells, plays an essential role in a variety of pathophysiological activities like cell division, differentiation, immune regulation and tumorigenesis (Zhang J. Q. et al., 2022). It has been reported that many upstream mediators can activate this pathway to exert their biological functions, comprising growth factors, hormones, and cytokines (Darnell et al., 1994; Liu J. et al., 2023). The JAK/STAT pathway consists of three parts: ligand-receptor complexes, JAKs, along with transcription factors STATs. Among the STAT protein family, STAT3 is the most well-studied member and its activation can play beneficial or detrimental roles in various diseases. On the one hand, STAT3 shows highly activated in most cancers and cardiac injuries (Xian et al., 2021; Zhuang et al., 2022) and is demonstrated to be a pathogenic regulator (Yu and Jove, 2004). On the other hand, STAT3 is also recognized as a protective molecule, and its activation may confer cardioprotection against several cardiovascular diseases including ischemia and ischemia-reperfusion injury (Negoro et al., 2000; Fuglesteg et al., 2008; Harhous et al., 2019) and cardiac hypertrophy (Enomoto et al., 2015). Recently, accumulating evidence has confirmed a novel profibrotic role of the JAK/STAT3 signaling activation in multiple tissues and organs, including the heart (Bao et al., 2020), liver (Ogata et al., 2006), kidney (Zheng et al., 2019), lung (Celada et al., 2018), and skin (Dees et al., 2020). In this regard, the JAK/STAT3 pathway may emerge as a potential therapeutic target for treating fibrotic diseases (Barry et al., 2007). However, there is a lack of a comprehensive summary on the role of the JAK/STAT3 signaling in mediating cardiac fibrosis. In this review, we discuss the structure, transactivation and regulation of the JAK/STAT3 signaling pathway and review current progress on the role of this pathway in cardiac fibrosis and challenges and opportunities of targeting the JAK/STAT3 signaling for the treatment of fibrosis.

## 2 The cellular and molecular mechanisms of cardiac fibrosis

Cardiac fibrosis usually occurs when myocardial tissue is suffering from a pathological stimulus such as ischemia, hypoxia, overload, inflammation or other pathogenic factors. It serves a dual role: it protects myocardial tissue integrity as a normal reparative response during injury, yet persistent and excessive scar formation greatly impairs the heart’s systolic and diastolic functions (Leask, 2015). Cardiac fibrosis not only increases ventricular stiffness but also induces the secretion of growth factors and cytokines to promote cardiomyocyte hypertrophy, ultimately leading to a decline in myocardial compliance, heart failure, and even sudden death (Mohammed et al., 2015; Francis Stuart et al., 2016).

Cardiac fibrosis is a common pathological feature manifested by multiple cardiovascular diseases, such as heart failure, hypertension, arrhythmia, cardiomyopathy, and myocardial infarction, and also plays a significant role in their onset and progression (Tao et al., 2014; Chen et al., 2015; Chung et al., 2021; Qi et al., 2022). Cardiac fibrosis manifests as the over-proliferation and differentiation of CFs and massive accumulation of extracellular matrix (ECM) components in the myocardium, like fibronectin, type I collagen, and type III collagen (Schafer et al., 2017). Myofibroblasts differentiated from CFs can synthesize contractile proteins like α-smooth muscle actin (α-SMA), leading to the distortion of tissue and cell structure (Hinz, 2007; Hinz, 2010). On the other hand, myofibroblasts can express excessive amounts of ECM proteins, thus leading to the substitution of permanent fibrotic scars for normal tissues, increased cardiac stiffness, and varying degrees of cardiac diastolic and systolic dysfunction (Weber, 1989; Cleutjens et al., 1995; Dobaczewski et al., 2006; Liu et al., 2017; Wang et al., 2022b).

The source of myofibroblasts in fibrotic hearts remains a disputed matter. Although some studies indicate that a significant proportion of myofibroblasts may originate from endothelial cells, epithelial cells or hematopoietic fibroblast progenitors (Möllmann et al., 2006; Zeisberg et al., 2007; Aisagbonhi et al., 2011), prevailing evidence confirms that the primary source of myofibroblasts in fibrotic heart tissue could be the activation of resident CFs (Ali et al., 2014; Moore-Morris et al., 2014; Kanisicak et al., 2016; Shinde and Frangogiannis, 2017; Moore-Morris et al., 2018). Furthermore, it has been suggested that pericytes could potentially serve as a reservoir of myofibroblasts, but the precise mechanism by which they operate remains uncertain, and there may be an overlap between pericytes and resident fibroblast subsets (Humphreys et al., 2010).

Although the molecular mechanisms involved in cardiac fibrosis are complex and variable, the transformation of CFs to myofibroblasts plays a central role in the process of cardiac fibrosis. Acute cardiac injury initiates a robust inflammatory response. This process involves the infiltration of immune cells into the cardiac tissue, which subsequently release inflammatory cytokines such as transforming growth factor (TGF)-β1, tumor necrosis factor-α (TNF-α) and interleukins (ILs) (Bujak and Frangogiannis, 2007; Christia et al., 2013). These cytokines activate CFs and instigate ECM remodeling through diverse signaling cascades. Concurrently, neurohormones within the renin-angiotensin-aldosterone system (RAAS) and the sympathetic nervous system, particularly Angiotensin II (Ang II), aldosterone, and catecholamines, are upregulated (Zou et al., 2004; Ferreira et al., 2016; Azushima et al., 2020). Their activation compels myofibroblasts to ramp up collagen production, culminating in the deposition of fibrotic tissue in the heart, which is a hallmark of cardiac remodeling. Additionally, mechanical stress, often a consequence of increased cardiac afterload in conditions like hypertension or valvular disease, prompts cardiomyocytes and fibroblasts to adapt by modifying their ECM, which alters their size, shape, and function (Li et al., 2018). Moreover, oxidative stress in the cardiac environment, primarily characterized by the overproduction of reactive oxygen species (ROS), inflicts direct cellular damage and fosters inflammation and apoptosis. These effects collectively trigger signaling pathways that exacerbate myocardial fibrosis (Grosche et al., 2018). Lastly, metabolic imbalances, including the production of advanced glycation end-products (AGEs) and lipotoxicity in cardiomyocytes, along with vascular implications like endothelial dysfunction, significantly contribute to the progression of cardiac fibrosis (Huby et al., 2015; Chen et al., 2016; Marciniec et al., 2017).

Among the aforementioned mediators, TGF-β1 is regarded as a central and potent profibrotic factor and evokes cardiac fibrosis mainly through activation of downstream classic small mother against decapentaplegic (Smad) signaling pathway. This process involves the binding of extracellular TGF-β1 ligand to TGF-β type II receptor (TGF-βRII), which phosphorylates TGF-β type I receptor (TGF-βRI). Activated TGF-βRI then phosphorylates and activates R-Smads (mainly Smad2 and Smad3), which further form a complex with Smad4. The complex moves to the nucleus and interacts with other co-activators to induce the transcription of fibrosis-related genes such as fibronectin, α-SMA and collagens (Shi and Massagué, 2003; Działo et al., 2018; Hu et al., 2018). Additionally, TGF-β1 also leads to cardiac fibrosis through activating several noncanonical (also called Smad-independent) signaling pathways, like phosphatidylinositol 3-kinase/protein kinase B (PI3K/Akt), mitogen-activated protein kinase [MAPK, mainly comprising p38, c-Jun NH_2_-terminal kinase (JNK) and extracellular signal-regulated kinase (ERK)] or Rho-like GTPases signaling pathways. In addition to the most common TGF-β signaling, the pathogenesis of cardiac fibrosis also involves a variety of other intracellular molecular pathways, including the JAK/STAT3 signaling (Zhang et al., 2019b), Wnt/β-Catenin signaling (Mizutani et al., 2016), integrin/focal adhesion kinase (FAK) signaling (Zhao et al., 2016; Molkentin et al., 2017), Hippo signaling (Singh et al., 2016), and myocardial related transcription factor (MRTF)/serum response factor (SRF) signaling (Tomasek et al., 2005; Lighthouse and Small, 2016). Therefore, targeting these fibrotic mediators or cascades could provide promising therapeutic approaches for treating fibrotic diseases.

## 3 Structure, function, transcriptional activity and regulation of the JAK/STAT3 signaling pathway

### 3.1 Molecular structure of STAT3

**FIGURE 1:**
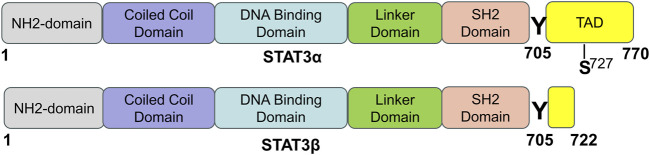
The domain structure and phosphorylation sites of STAT3 protein. STAT3 has two splicing isoforms, STAT3α and STAT3β, and they are comprised of 770 and 722 amino acids, respectively. STAT3 contains six different functional domains, including the NH_2_-terminal domain, coiled-coil domain, DNA binding domain, linker domain, SH2 domain, and COOH-terminal transactivation domain (TAD). “Y” means a tyrosine phosphorylation site, and “S” means a serine phosphorylation site [adapted from ref. (Hu et al., 2021).

In mammals, there are seven proteins belonging to the STAT family, which consists of cytoplasmic transcription factors named STAT1-STAT4, STAT5a, STAT5b, and STAT6 (Hu et al., 2020b). Among these, STAT3 is the most extensively studied and plays pivotal roles in controlling various cellular biological processes. STAT3 was originally discovered in 1994 through a series of studies on cytokine-induced acute responses of target genes. Unlike other family members, global deletion of STAT3 can cause embryonic death. The STAT3 protein consists of 770 amino acid residues and, similar to other members of the STAT family, it can be divided into six distinct functional domains (Figure 1): an NH_2_-terminal domain (NTD), a coiled-coil domain (CCD), a DNA binding domain (DBD), a linker domain (LD), an Src homology 2 (SH2) domain, and a COOH-terminal transactivation domain (TAD). Each domain has a specific function (Hu et al., 2021) (Table 1).

**TABLE 1:** Function of STAT3 domains.

Domain	Function Kishore and Verma (2012), Haghikia et al. (2014), Harhous et al. (2019), Hu et al. (2021)
NTD	Promoting the formation of STAT3 dimers and regulating nuclear translocation
CCD	Providing binding sites for regulatory factors and participating in regulating nuclear import and export
DBD	Recognizing and binding to specific DNA elements of target genes
LD	Affecting DNA binding stability
SH2	Recognizing phosphotyrosine sites of receptors and contributing to form STAT3 dimers
TAD	Recruiting co-activators and regulating target gene transcription

**Abbreviation:** NTD, NH_2_-terminal domain; CCD, coiled-coiled domain; DBD, DNA-binding domain; LD, linker domain; SH2, Src homology 2 domain; TAD, COOH-terminal transactivation domain.

STAT3 is expressed widely in different cell types within the heart, such as cardiomyocytes, fibroblasts, immune cells, and endothelial cells. Two isoforms of the STAT3 protein, STAT3α (92 kDa) and STAT3β (83 kDa), are produced through alternative splicing of the identical gene. STAT3β is missing the COOH-terminal 55 amino acids, which are correspondingly replaced by seven distinct amino acid residues (Schaefer et al., 1995; Caldenhoven et al., 1996). Research has shown that while STAT3β is not vital for survival, mice deficient in STAT3α do not survive past birth (Maritano et al., 2004). STAT3α possesses two phosphorylation sites, namely, Tyr705 and Ser727, whereas STAT3β only possesses one phosphorylation site, specifically Tyr705. When either Tyr705 or Ser727 is phosphorylated, STAT3 is activated and exerts its function. STAT3 can be activated by more than 50 extracellular ligands, which are commonly some cytokines, hormones, growth factors, and chemokines, such as ILs, interferons, colony-stimulating factors, epidermal growth factor (EGF), and platelet-derived growth factor (PDGF) (Darnell, 1997; Hu et al., 2021). STAT3’s biological functions are complicated and diverse and its main physiological roles under normal conditions are summarized in the following section.

STAT3 is an important intracellular signaling molecule that has multiple functions under normal physiological conditions. These functions include: (1) Regulating the proliferation and differentiation of various cell types by binding to specific DNA sequences and affecting gene expression. For example, STAT3 promotes the proliferation of corneal limbal keratinocytes via a ΔNp63-dependent mechanism, and inhibiting this pathway can increase cell differentiation (Hsueh et al., 2011). STAT3 also mediates megakaryocyte differentiation induced by RAD001 (Su et al., 2013). (2) Regulating the activation, proliferation, and secretion of cytokines by immune cells, which can modulate immune responses and inflammation. For instance, STAT3 inhibition can induce apoptosis and/or activate effective immune responses in colon cancer cells, overcoming cancer-induced immune tolerance (Jahangiri et al., 2020). Likewise, systemic injection of penetrating c-Myc and gp130 peptides can inhibit pancreatic tumor growth and induce anti-tumor immunity (Aftabizadeh et al., 2021). (3) Mediating the expression of inflammation-related genes in response to various cytokines and growth factors. One of the most prominent examples is IL-6, which we will discuss in detail later. (4) Maintaining the self-renewal and differentiation of stem cells by regulating the transcription of target genes. Phosphorylated STAT3 is functionally associated with the expression of self-renewal genes in embryonic stem cells (Bourillot et al., 2009). Moreover, constitutively activated STAT3 can sustain the self-renewal process in the absence of leukemia inhibitory factor (LIF) (Matsuda et al., 1999). (5) Participating in tissue repair and regeneration processes by modulating cell survival and growth. For instance, Transmembrane and ubiquitin like domain containing 1 (Tmub1) inhibits the phosphorylation and activation of STAT3, impairing liver regeneration in mice after partial hepatectomy (Fu et al., 2019). Conversely, Krüppel-like factor 4 (KLF4) deletion *in vivo* induces axonal regeneration in adult retinal ganglion cells (RGCs) through the JAK/STAT3 signaling pathway. This regeneration can be further enhanced by removing the endogenous JAK/STAT3 pathway inhibitor SOCS3 (Qin et al., 2013). (6) Regulating the energy metabolism of cells by influencing the expression of mitochondrial oxidative phosphorylation-related genes. For example, icaritin inhibits the survival and glycolysis of glioblastoma (GBM) cells through the IL-6/STAT3 pathway (Li et al., 2019a). Additionally, STAT3 promotes mitochondrial respiration and reduces the production of ROS in neural precursor cells (Su et al., 2020). (7) Playing an essential role in early embryonic development, as embryos with STAT3 gene defects will die in the early stages of development. In humans, LIF and STAT3 are expressed in decidual tissue during early pregnancy. LIF can induce STAT3 phosphorylation in non-decidualized and decidualized human endometrial stromal cells *in vitro*, suggesting that LIF/STAT3 signaling is involved in human embryo implantation and decidualization (Shuya et al., 2011). Furthermore, conditional ablation of STAT3 in the uterus can result in embryo implantation failure (Lee et al., 2013).

### 3.2 Molecular structure of JAK

In mammals, the JAK family consists of four main members (JAK1-JAK3 and Tyk2), which are non-receptor tyrosine protein kinases (Schindler and Darnell, 1995). JAK1, JAK2, and Tyk2 have broad expression, whereas JAK3 is mainly present in cells of the hematopoietic lineage (Speirs et al., 2018). Upon interaction of cytokines or growth factors with their corresponding receptors, JAK tyrosine kinases are activated, thereby facilitating intracellular signal transduction.

**FIGURE 2:**
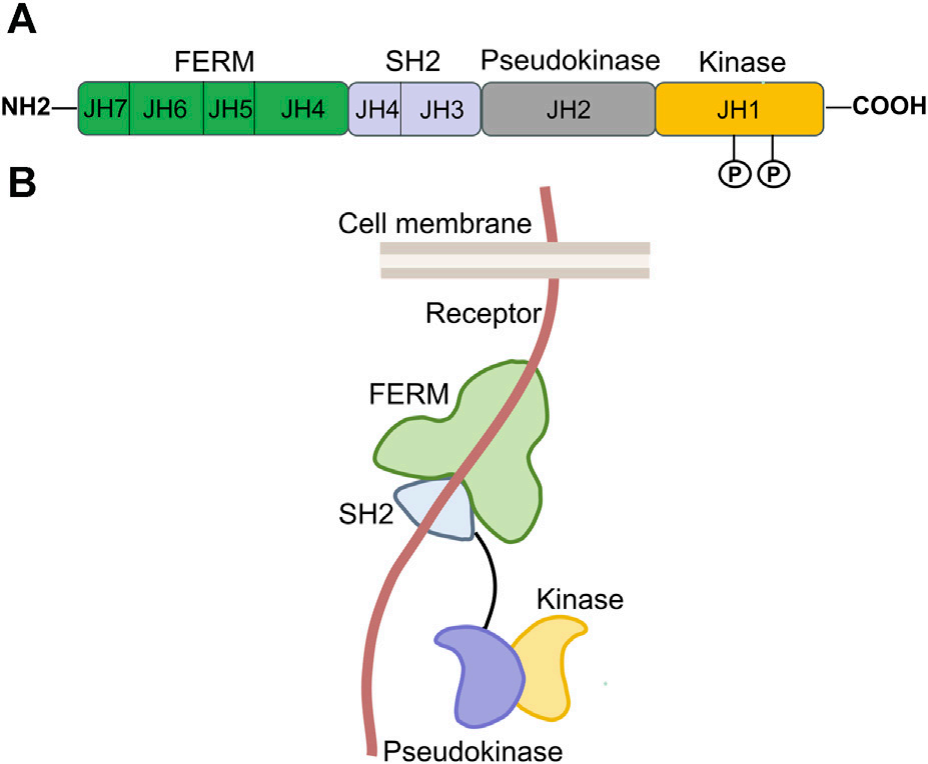
Structure of JAK. **(A)**. Domains and conserved phosphorylation sites of the JAK protein. The JAK protein family contains four members, JAK1-3, and TYK2. Each is composed of seven homologous regions, labeled JH1-JH7. These regions make up four distinct functional domains, of which, JH1 corresponds to the kinase domain; JH2 is the pseudokinase domain; JH3 and a portion of JH4 together form the SH2 domain; and the combination of JH5, JH6, JH7, and the rest of JH4 constitutes the FERM domain. “P” represents conserved tyrosine phosphorylation sites of the JAK protein. **(B)**. Three-dimensional spatial structure of JAK in cells [adapted from ref. (Hu et al., 2021).

The JAK protein is made up of seven similar regions (JH1-JH7) and includes four functional domains: a domain for tyrosine kinase, a domain for pseudokinase, an SH2 domain, and an NH2-terminal FERM domain (Four-point-one protein, Ezrin, Radixin, Moesin) (Figure 2) (Banerjee et al., 2017). The carboxy-terminal portion of each JAK includes the catalytic kinase domain (JH1) and the pseudokinase domain (JH2). JH1, containing nearly 250 amino acid residues, is the active phosphotransferase domain needed for phosphorylation of cytokine receptors and downstream STAT proteins. JH2 is similar to JH1 in structure, but it is generally considered to have no catalytic activity and can regulate the kinase activity of JH1 (Zhao et al., 2018; Xin et al., 2020). According to reports, the JAK2 protein’s JH2 exhibits a minimal level of kinase activity as stated by Ungureanu et al. (2011). The N-terminal region of each JAK contains the SH2 (JH3 with half of JH4) and FERM (JH5-JH7 and one-half of JH4) domains, which collectively facilitate the interaction between JAK proteins and the box1/2 regions of cytokine receptors located near the cell membrane (Saharinen et al., 2000; Wallweber et al., 2014; Hubbard, 2017; Morris et al., 2018; Xin et al., 2020; Raivola et al., 2021).

### 3.3 Canonical JAK/STAT3 signaling pathway

**FIGURE 3:**
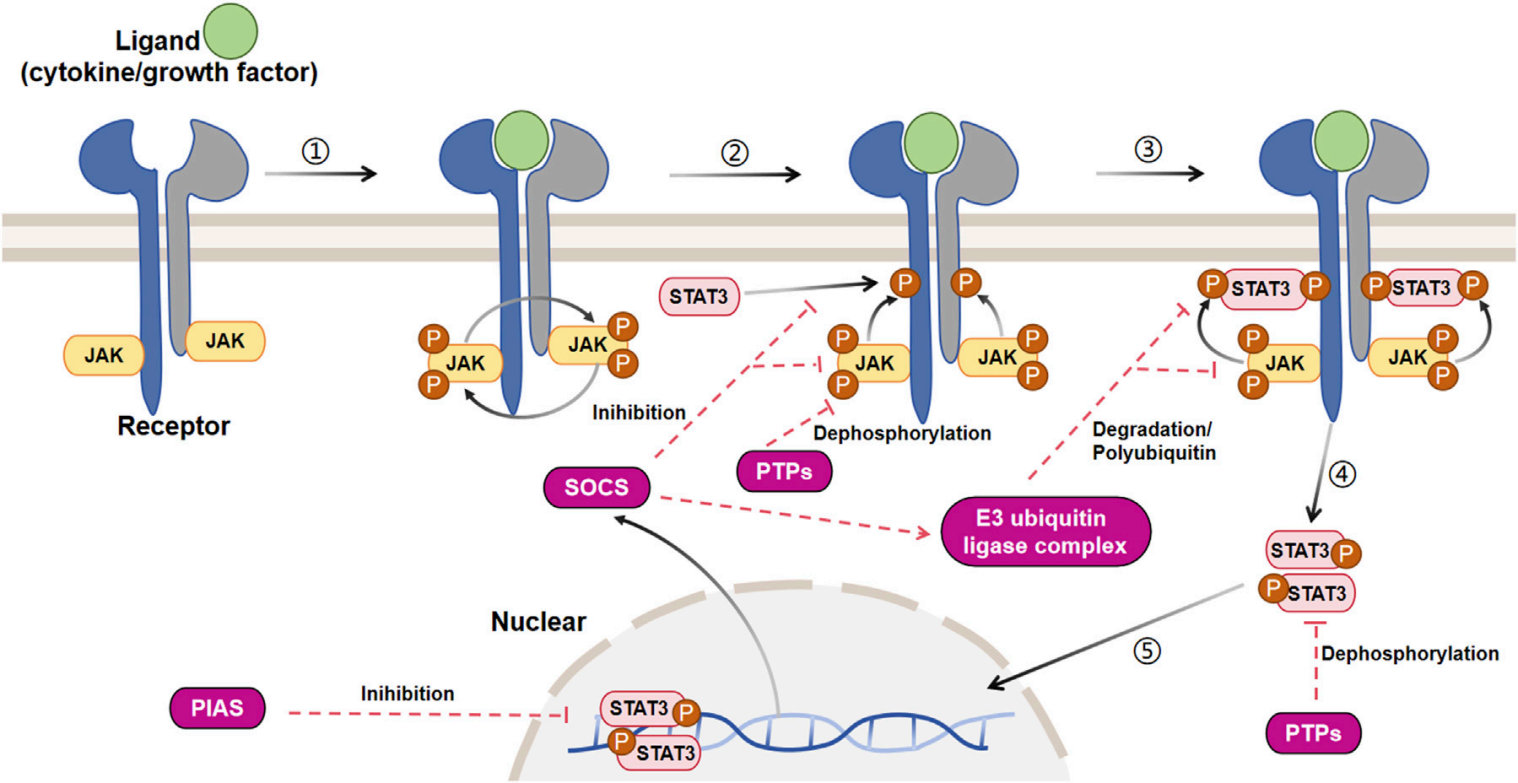
Signal transduction and negative regulation of the canonical JAK/STAT3 pathway. The JAK/STAT3 cascade is initiated by the interaction between a ligand and its corresponding receptor. This interaction leads to the auto-phosphorylation of the JAK kinase bound to the receptor. Once activated, JAK phosphorylates a tyrosine residue on the receptor, creating a docking site for cytoplasmic STAT3 and recruiting STAT3. At this docking site, JAK phosphorylates STAT3. The phosphorylated STAT3 then dissociates from the receptor and forms dimers. These STAT3 dimers move to the nucleus, where they bind to promoters and regulate transcription. The JAK/STAT3 cascade is controlled by three primary types of negative regulators: PTPs (protein tyrosine phosphatases), PIAS (protein inhibitor of activated STAT), and CIS/SOCS (suppressor of cytokine signaling). PTPs block the JAK/STAT3 signaling mainly by interacting directly with the STAT3 dimers and JAK to dephosphorylate them. PIAS prevents the JAK/STAT3 signaling principally by inhibiting the binding of STAT3 to DNA. As a common objective caused by the activation of JAK/STAT3, CIS/SOCS mainly hinders the JAK/STAT3 cascade through the following methods: (1) obstructing the recruitment of STAT3 to the phosphorylated receptor; (2) directly interacting with JAK to suppress its kinase function; (3) prompting the creation of an E3 ubiquitin ligase complex that breaks down JAK or prevents STAT3 from binding to the SOCS protein [adapted from refs. (Gurzov et al., 2016; Hu et al., 2021).

The JAK/STAT signaling pathway is activated by more than 50 cytokines and growth factors, including hormones, interferons (IFN), ILs, and colony stimulating factors (Darnell, 1997). These molecules regulate various cellular events, such as hematopoiesis, immune adaptability, tissue repair, inflammation, cell apoptosis, and adipogenesis (Owen et al., 2019). The JAK/STAT3 pathway is activated when these extracellular ligands bind to their dedicated transmembrane receptors (Figure 3). The cytosolic domains of these receptors are constitutively interacting with receptor-related JAK tyrosine kinases. These JAK kinases are nonactivated before the ligand stimulation, while the coupling of the ligand with its receptor results in auto-phosphorylation of JAK kinases (Feng et al., 1997). Upon activation, the JAK molecules phosphorylate the cytoplasmic segment of the receptors at particular tyrosine residues, subsequently serving as binding sites for cytoplasmic STAT3 protein and attracting the recruitment of the STAT3 protein. After docking, STAT3 is phosphorylated by JAK kinase and subsequently associates with itself or other phosphorylated STAT monomers to create homodimers or heterodimers upon separation from the receptor. Ultimately, these dynamic molecular pairs migrate from the cytoplasm to the nucleus, where they attach to target gene promoters and stimulate the expression of target genes (O'Shea et al., 2015; Durham et al., 2019), often causing proliferation, differentiation, and apoptosis.

### 3.4 Noncanonical JAK/STAT3 signaling pathway

The function of STAT3 is influenced by different post-translational modifications, including phosphorylation, methylation, acetylation, and ubiquitination, occurring at various amino acid sites. In addition to classical signal transduction, JAK/STAT3 may also play a role in nonclassical signal transduction. Research has indicated that STAT3, which is not phosphorylated on Tyr705, has the ability to move from cytoplasm to the nucleus and can activate various STAT3 target genes in the absence of Ser727 phosphorylation (Bharadwaj et al., 2020). Additionally, the process can be facilitated by Lys685 acetylation and NF-kB signaling activation, as suggested by previous studies (Yang et al., 2007; Dasgupta et al., 2014). Besides being activated in the cytosol, all STAT proteins (excluding STAT4) have the ability to localize to the mitochondrion, leading to an enhancement in oxidative phosphorylation and membrane polarization. For example, STAT3 monomers phosphorylated on Ser727 can translocate into the mitochondrion without dimerization to increase membrane polarization and ATP synthesis, and inhibit ROS production and mitochondrial permeability transition pore (MPTP) opening, thus exerting a protective role (Boengler et al., 2010; Garama et al., 2016; Avalle and Poli, 2018). Besides, STAT3 has also been reported to translocate to the endoplasmic reticulum and contribute to reduce oxidative stress-induced apoptosis (Avalle et al., 2019). In the nucleus, certain STAT molecules that are not phosphorylated interact with heterochromatin protein 1 (HP1) located on heterochromatin. Phosphorylation of STAT by JAK or other kinases can cause the detachment of HP1 from heterochromatin, leading to its destabilization. Subsequently, phospho-STAT can interact with particular regions on autosomes and regulate the expression of target genes (Shi et al., 2006; Shi et al., 2008b; Li, 2008). This noncanonical JAK/STAT signaling is critical for sustaining heterochromatin stability. Moreover, increasing evidence has shown that activation of JAK/STAT signaling can cause chromatin remodeling in mammals (Christova et al., 2007; Shi et al., 2008a). Besides being triggered by JAK, STAT3 can also be activated by alternative non-receptor tyrosine kinases or JAK-independent receptors. As an example, the c-Src enzyme is capable of phosphorylating STAT3, which then can promote the expression of oncogenes (Yu et al., 1995). EGF receptor and PDGF receptor can directly activate STAT3 (Ruff-Jamison et al., 1994; Liu et al., 2023a).

### 3.5 Cross-talk between the STAT3 signaling and other pathways

Besides the prevalent JAK/STAT3 signaling pathway, STAT3 also engages in alternative signaling pathways or establishes communication with these pathways, thereby producing biological impacts. STAT3 is involved in the classic TGF-β/Smad signaling pathway (Pedroza et al., 2018; Chen et al., 2019b; Sun et al., 2022) and Smad-independent TGF-β signaling pathways, such as the ERK-mediated MAPK (Park et al., 2020; Shen et al., 2021), JNK (Park et al., 2020), and PI3K/Akt signaling pathways (Zhu et al., 2018; Lee et al., 2019). In addition to TGF-β-related signaling pathways, STAT3 also participates in many other signaling cascades, such as Fyn (a member of the Src kinase family) (Seo et al., 2016; Zhu et al., 2018; Zhu et al., 2023), peroxisome proliferator-activated receptor (PPAR) (Lo et al., 2017b; Németh et al., 2019), and Notch signaling (Chen et al., 2019c).

### 3.6 Negative regulation of canonical JAK/STAT3 signaling

The inhibition of canonical JAK/STAT3 signaling involves three primary categories of negative regulators (Figure 3): protein inhibitor of activated STAT (PIAS), protein tyrosine phosphatases (PTPs), and suppressor of cytokine signaling (SOCS/CIS). These regulators, as described by Liongue et al., play a crucial role in preventing the excessive phosphorylation of STAT3 (Liongue et al., 2016; Villarino et al., 2017; Yang et al., 2017).

The process of JAK/STAT signal transduction contains a series of intracellular tyrosine phosphorylation, so PTPs have a key role in regulating this pathway. PTPs can directly dephosphorylate and inactivate the STAT dimers, and block the JAK/STAT cascade. For instance, a receptor tyrosine phosphatase PTPRTR can bind to and dephosphorylate the tyrosine residue at site 705 in STAT3 (Zhang et al., 2007). SHP-2, a significant member of the PTP family and also a target gene for activated STAT3, can decrease the phosphorylation level of STAT3 (Schmitz et al., 2000). In addition, PTPs can dephosphorylate JAK and prevent the JAK/STAT signaling.

The PIAS family comprises four transcription regulatory factors, namely, PIAS1-PIAS4. PIAS was originally identified to be a suppressor of STAT, and PIAS3 can combine with STAT3. PIAS only binds to phosphorylated STAT dimers rather than STAT monomers (Hu et al., 2021). PIAS mainly suppresses the transcriptional activity of STAT by means of three mechanisms. (1) Preventing the DNA-binding activity of STAT and blocking STAT-DNA interactions (Sonnenblick et al., 2004). (2) Recruiting transcriptional co-inhibitory factor such as histone deacetylase (Tussié-Luna et al., 2002). (3) Promoting STAT SUMOylation (Yuan et al., 2015).

SOCS family proteins are considered as major triggers of the JAK/STAT signaling attenuation, and there are eight members in this family: SOCS1-7 and cytokine-inducible SH2 protein (CIS) (Minamoto et al., 1997; Piessevaux et al., 2008; Kazi et al., 2014). Cytokine-stimulated JAK/STAT signaling activation induces the SOCS proteins, which act as negative feedback suppressors to regulate this pathway (Naka et al., 1997; Kershaw et al., 2013b). For example, SOCS3 gene is quickly induced by phosphorylated STAT3 dimers in the nucleus, and in turn SOCS3 protein interacts with activated JAK and its receptor to suppress JAK activity, thus preventing further JAK/STAT3 signaling activation (Babon et al., 2012; Kershaw et al., 2013a). SOCS primarily inhibits the JAK/STAT cascade in the following ways. (1) It competes with STAT for binding to the phosphorylated receptor and prevents STAT recruitment. (2) It forms an E3 ubiquitin ligase complex via the COOH-terminal SOCS box and degrades JAK or STAT that binds to SOCS (Kamran et al., 2013). (3) The SOCS protein has the ability to directly and specifically interact with either JAK or its receptor in order to inhibit the activity of JAK kinase. An example is the presence of a distinct brief pattern known as the kinase inhibitory region (KIR) in SOCS1 and SOCS3. This pattern enables these two proteins to hinder the catalytic activity of JAK by directly binding to JAK or its receptor (Sasaki et al., 1999; Yasukawa et al., 1999; Alexander, 2002).

### 3.7 The JAK/STAT3 pathway induces fibrosis

**FIGURE 4:**
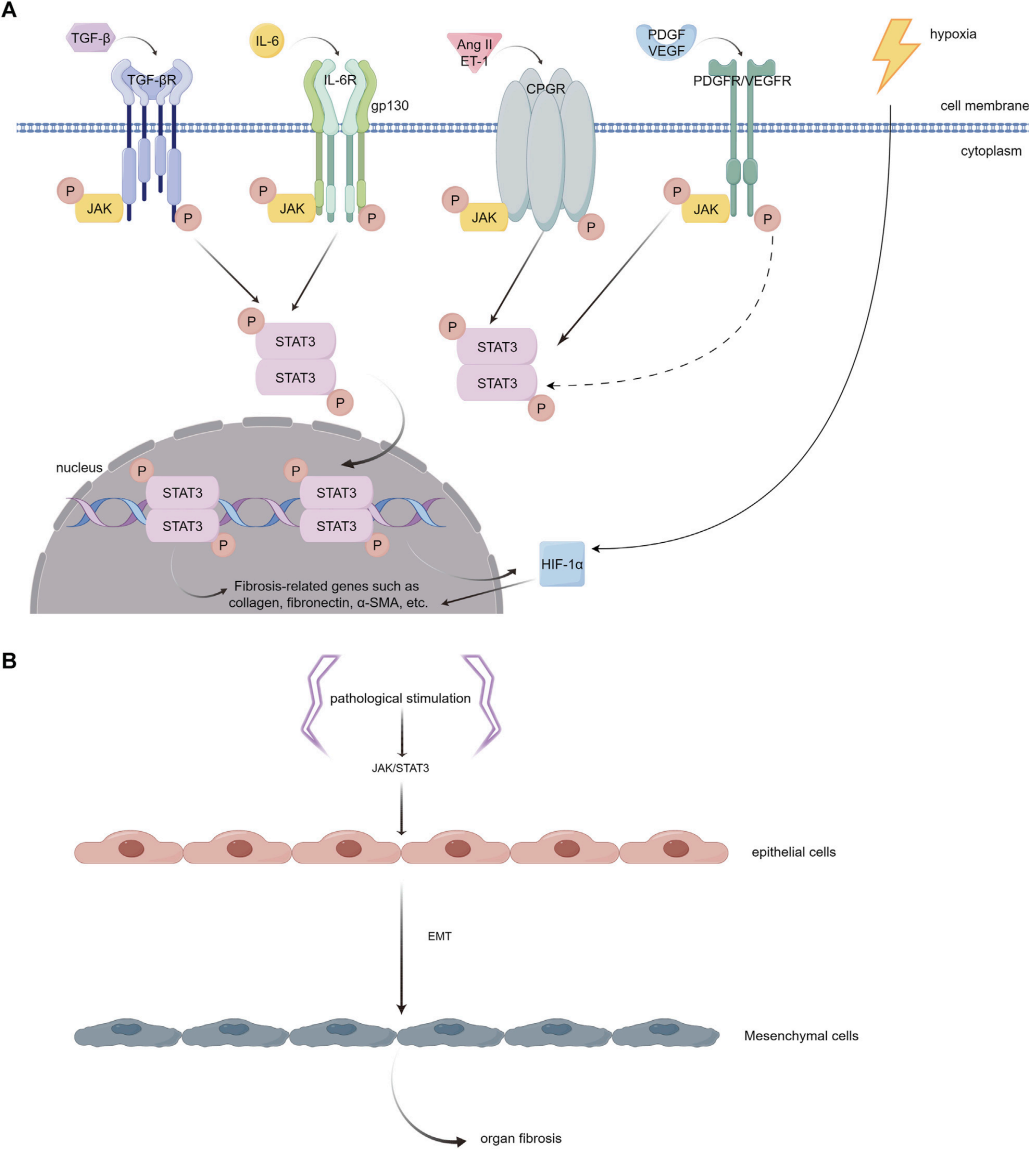
**(A)**. Different JAK/STAT3 activators that play important roles in the pathophysiology of myocardial fibrosis. (1) TGF-β interacts with its receptor (TGF-βR) on the cell surface, initiating receptor kinase activity. This activity leads to JAK phosphorylation and subsequent activation of STAT3. However, the precise mechanism underlying this process remains to be fully elucidated. (2) IL-6 binds to its specific receptor, IL-6R, forming a complex. This complex then associates with the membrane protein gp130. Activation of JAKs, which are associated with gp130, is critical for phosphorylating specific tyrosine residues on gp130. These residues act as anchoring points for STAT3. (3) Ang II and ET-1 engage with the GPCR family, triggering the phosphorylation of tyrosine in JAK kinase and consequently activating STAT3. (4) PDGF and VEGF each bind to their respective tyrosine kinase receptors. This binding results in the phosphorylation of tyrosine residues on the receptors, which can indirectly or transactivate JAK, leading to the activation of the STAT3 pathway. Once phosphorylated, STAT3 dimerizes and moves into the nucleus. In the nucleus, these STAT3 dimers attach to specific DNA sequences, enhancing the transcription of genes that are pivotal in driving inflammation and fibrosis, including collagen, fibronectin, α-SMA, etc. In addition, the activation of STAT3 has the capability to stimulate the expression of HIF-1α and enhance the production of ECM in hypoxic environments. **(B)**. Epithelial to mesenchymal transition (EMT). The activation of JAK/STAT3 signaling by pathological stimuli has the potential to induce a phenotypic transition of epithelial cells into mesenchymal cells. These mesenchymal cells exhibit enhanced migration and invasion capabilities. (By Figdraw).

Studies have indicated that the JAK/STAT3 pathway plays a key role in the process of fibrosis. It can be activated by various pro-fibrotic mediators, such as TGF-β1, PDGF, vascular endothelial growth factor (VEGF), IL-6, Ang II, serotonin (5-HT), and endothelin (ET-1), and then leads to fibrogenesis (Rane and Reddy, 2000; Zhang et al., 2015; Roskoski, 2016) (Figure 4A). The JAK/STAT3 pathway is also demonstrated to be a central integrator of multiple pro-fibrotic pathways and its activation can promote the activation of fibroblasts and the expression of fibrosis-related genes, such as α-SMA, collagens, and fibronectin (Zhang et al., 2015; Chakraborty et al., 2017; Dees et al., 2020). In addition, once activated, STAT3 can induce the expression of hypoxia-inducible factor-1α (HIF-1α), a transcription factor that responds to hypoxic conditions and stimulates the production of ECM (Yang et al., 2021) (Figure 4A). Activated STAT3 can also trigger epithelial to mesenchymal transition (EMT), a cellular process that allows epithelial cells to transform into mesenchymal cells with more power in migration and invasion, and facilitates the progression of fibrosis (Montero et al., 2021; Yang et al., 2021) (Figure 4B).

### 3.8 The effects of the JAK/STAT3 pathway on different types of cardiac injury

**FIGURE 5:**
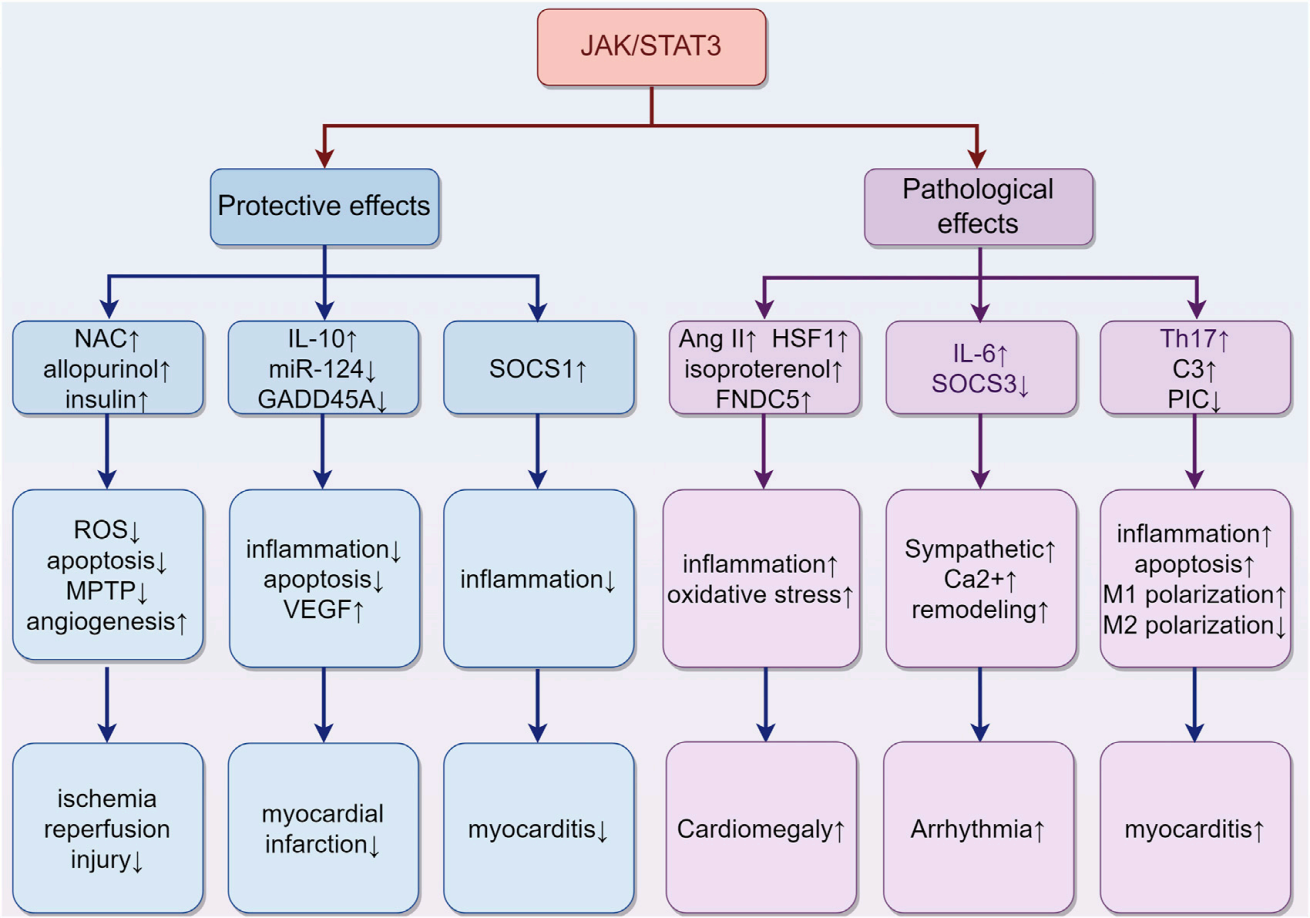
The role of activation of the JAK/STAT3 pathway in different types of cardiac damage. (1) In ischemia-reperfusion injury, agents such as NAC, allopurinol, and insulin may confer protective effects. They achieve this by reducing ROS production and cardiomyocyte apoptosis, promoting angiogenesis, and delaying the opening of the MPTP. (2) In the case of myocardial infarction, certain molecular factors like miR-124, IL-10, and GADD45A exert beneficial effects through the STAT3 pathway. These include anti-apoptotic (miR-124), anti-inflammatory (IL-10), and VEGF-mediated angiogenic effects (GADD45A), collectively contributing to improved prognosis. (3) The situation of myocarditis is more complex. The upregulation of SOCS1 can inhibit inflammation. Meanwhile, the upregulation of complement C3 and Th17 cells, along with the downregulation of Piceatannol, may exacerbate inflammation. These findings highlight the multifaceted impact on the progression of myocarditis. (4) Cardiac hypertrophy is influenced by Ang II, HSF1, isoproterenol, and FNDC5, which collaboratively induce hypertrophy through increased oxidative stress and inflammation. (5) Arrhythmias are closely associated with JAK/STAT3 activity, which contributes to myocardial sarcoplasmic reticulum Ca2+ overload, increased cardiac sympathetic nerve activity, and ventricular remodeling. “↑” represents activation, upregulation or exacerbation, and “↓” represents inhibition, downregulation or relief. (By Figdraw).

The JAK/STAT3 pathway plays a pivotal role in various aspects of cardiac physiology and pathology, exhibiting multifaceted roles in the heart (Figure 5). It mediates protective effects in different stages of ischemia, including ischemia pre-, post-, and remote conditioning (Hattori et al., 2001; You et al., 2011; Gao et al., 2017). Agents such as N-acetylcysteine (NAC) and allopurinol (Wang et al., 2013), and insulin (Fuglesteg et al., 2008) are known to protect against myocardial ischemia-reperfusion injury through activation of the JAK/STAT3 pathway. Their protective mechanism likely involves the reduction of ROS production, decrease in cardiomyocyte apoptosis, promotion of angiogenesis, and delay in MPTP opening. In the context of myocardial infarction, molecular factors like miR-124, IL-10, and growth arrest and DNA damage-inducible α (GADD45A) exert beneficial effects through the STAT3 pathway. Specifically, miR-124 offers anti-apoptotic benefits, IL-10 provides anti-inflammatory effects, and GADD45A enhances VEGF-mediated angiogenesis, collectively improving prognosis (He et al., 2018; Wang et al., 2022a; Tesoro et al., 2022). Conversely, conditional deletion of STAT3 in cardiomyocytes exacerbates cardiac remodeling during the subacute phase of myocardial infarction or under chronic β-adrenergic stimulation (Enomoto et al., 2015; Zhang et al., 2016). Furthermore, cardiomyocyte-specific transgenic expression of SOCS1 inhibits JAK/STAT3 activation in enterovirus-induced myocarditis, but this is associated with increased mortality in mice, highlighting a complex interplay (Yasukawa et al., 2003).

Despite its protective roles, the JAK/STAT3 pathway also has detrimental effects. For instance, in myocarditis, IL-6-triggered increases in liver complement C3 and Th17 cells may exacerbate inflammation (Camporeale et al., 2013; Wang et al., 2020). Additionally, inhibiting the JAK/STAT3 signaling with piceatannol could improve sepsis-induced cardiac dysfunction by relieving cell apoptosis and inflammation in septic mice and H9C2 cardiomyocytes, suggesting a critical role of the JAK/STAT3 pathway in sepsis-related myocardial injury (Xie et al., 2021). This pathway also skews macrophage polarization towards M1 and away from M2, contributing to coxsackievirus B3 (CVB3)-induced myocardial inflammation and injury (Wang et al., 2023). Chronic activation of JAK/STAT3 can induce cardiac hypertrophy, as evidenced by Ang II-induced activation of TLR4 and STAT3, promoting hypertrophy via the IL-6/JAK2/STAT3 pathway (Han et al., 2018). Other activators like Heat-shock transcription factor 1 (HSF1), isoproterenol, and Fibronectin type III domain containing 5 (FNDC5) also trigger this pathway, resulting in increased cardiac inflammation, oxidative stress, and pathological hypertrophy (Zhao et al., 2017; Yuan et al., 2018; Geng et al., 2019). Moreover, JAK/STAT3 is implicated in cardiac arrhythmias. Inhibiting JAK2/STAT3 phosphorylation reduces malignant ventricular arrhythmias post-myocardial infarction by attenuating ventricular remodeling (Gao et al., 2020). Cardiac-specific SOCS3 gene knockout mice exhibit myocardial sarcoplasmic reticulum Ca^2+^ overload and subsequent ventricular arrhythmias because of the activation of cardiac gp130 signaling (Yajima et al., 2011). Additionally, IL-6 overexpression, via the STAT3 pathway, promotes cardiac sympathetic nerve activity, increasing the incidence of ventricular arrhythmias (Peng et al., 2023).

## 4 Multiple mediators regulate cardiac fibrosis through the STAT3 signaling pathway

### 4.1 ILs

ILs are a type of cytokine proteins that various cells, mainly immune ones, produce. Cytokines modulate cellular functions such as growth, maturation, movement, adhesion, activation and differentiation (Zhang and An, 2007; Brocker et al., 2010). ILs are a large family of cytokines with more than 60 members, which can be grouped into four categories: IL-1 related, type 1 helical (IL-4 related, γ chain and IL-6/IL-12 related), type 2 helical (IL-10 related and IL-28 related), and IL-17 related (Brocker et al., 2010). ILs regulate homeostasis by influencing the cardiovascular, neuroendocrine and metabolic systems in the human body (Corwin, 2000).

**FIGURE 6:**
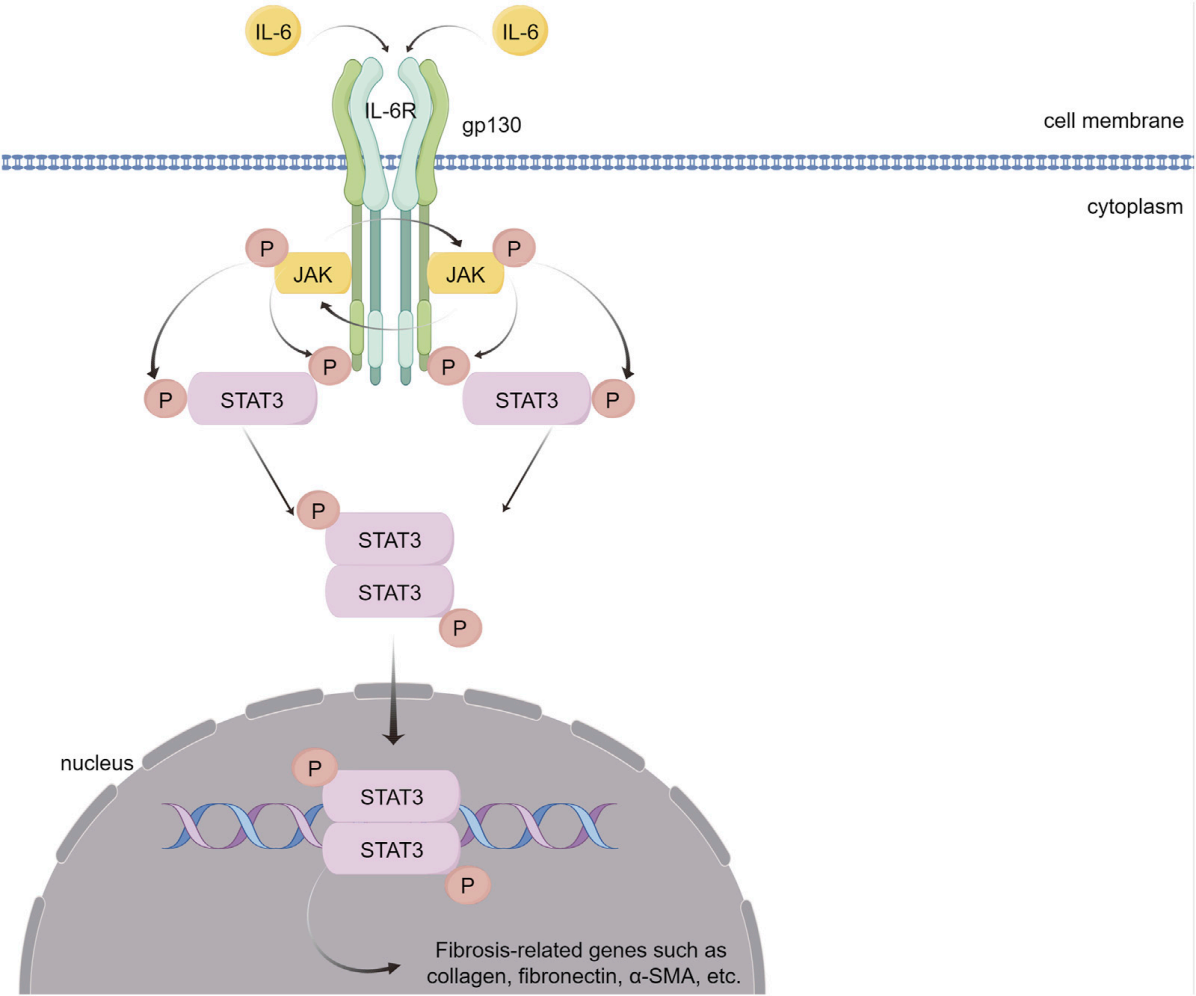
IL-6 causes myocardial fibrosis through the JAK/STAT3 signaling pathway. IL-6 binds to its receptor, IL-6R, forming a complex that activates the gp130 receptor. This activation triggers the JAK family of tyrosine kinases. Once activated, these JAKs phosphorylate STAT3, a crucial step in the signaling pathway. Phosphorylated STAT3 dimerizes and translocates into the nucleus. There, STAT3 dimers bind to specific DNA sequences, promoting the transcription of genes that are pivotal in mediating inflammation and fibrosis. (By Figdraw).

Recent research has demonstrated that ILs contribute to myocardial fibrosis via the STAT3 pathway. Some ILs play proinflammatory and fibrotic roles, and IL-6 is the most representative (Figure 6). In the absence of NF-E2-related factor 2 (Nrf2), IL-6 levels further increase in response to Ang II, thereby activating the IL-6/STAT3 pathway, which causes cardiomegaly and inflammation (Chen et al., 2019a). In addition, Ang II can induce Toll-like receptor phosphorylation of STAT3, increase IL-6 production, and continuously activate the JAK/STAT pathway, thereby providing positive feedback and promoting myocardial hypertrophy, fibrosis, and ventricular remodeling (Chen et al., 2017a; Han et al., 2018; Zhang et al., 2019b). IL-6 enhances STAT3 phosphorylation in cultured CFs, whereas inhibiting STAT3 reduces IL6-induced collagen synthesis and reverses pressure overload-induced cardiac hypertrophy (Mir et al., 2012). In a transverse aortic constriction (TAC)-induced mouse heart failure model, inhibiting IL6/gp130/STAT3 with raloxifene alleviated TAC-induced myocarditis, cardiac remodeling and dysfunction (Huo et al., 2021). In mice with CVB3-induced dilated cardiomyopathy (DCM), IL-6 knockout reduced the phosphorylation level of STAT3 in myocardial tissue, thereby improving myocardial remodeling induced by DCM (Li et al., 2019b).

### 4.2 TGF-β

**FIGURE 7:**
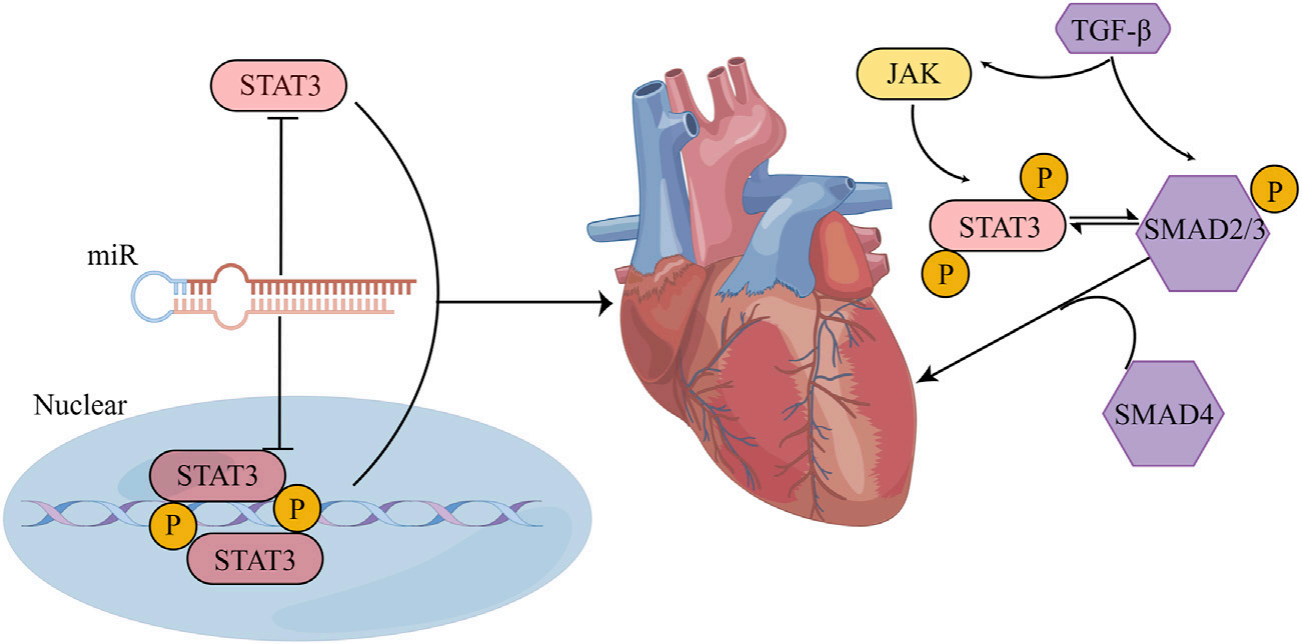
STAT3 influences cardiac fibrosis through multiple pathways. (1) The crosstalk between STAT3 and miR manifests in several ways: STAT3 can form either a direct feedback or an indirect feedback loop by binding with miR; it can also mediate the transcription of downstream miR; meanwhile, miR can influence the translation of STAT3 mRNA. (2) Positioned downstream of the TGF-β/SMAD signaling cascade, STAT3 might collaboratively regulate myocardial fibrosis with TGF-β. Their synergistic action could potentially be associated with the phosphorylation of STAT3. (By Figdraw).

The TGF-β and STAT3 signaling pathways have a feedback loop that regulates the acute/chronic stress response in the heart. TGF-β signaling affects STAT3 as an important target in its downstream pathway (Pedroza et al., 2018; Chen et al., 2019b; Sun et al., 2022). Several studies have demonstrated the interaction between TGF-β and STAT3 in cardiac fibrosis. For instance, it has been reported that TGF-β-induced CD44/STAT3 signaling plays a crucial part in atrial fibrosis and fibrillation formation. CD44 is a membrane receptor that modulates fibrosis. Blocking CD44 signaling can reduce TGF-β-induced STAT3 activation and collagen expression in atrial fibroblasts, implicating a potential approach for treating atrial fibrosis and fibrillation (Chang et al., 2017). Moreover, Ephrinb2-mediated myocardial fibrosis involves the activation of the TGF-β/Smad3 and STAT3 pathways. Further study revealed that Ephrinb2 could enhance the interaction of TGF-β/Smad3 and STAT3 signaling to promote cardiac fibrosis (Su et al., 2017). Furthermore, tyrosine mutation at site 705 to glutamic acid constitutively activated STAT3, which could further enhance the interaction between Smad3 and STAT3 (Su et al., 2017). One previous study showed that a high-fat diet could activate the left ventricular renin–angiotensin system (RAS) and JAK1/2-STAT1/3 pathways in rats by increasing ROS and IL-6 production, ultimately causing cardiac fibrosis. This creates a positive feedback loop that activates the TGF-β1/Smad3 fibrotic pathway and enhances left ventricular collagen synthesis (Eid et al., 2019). In cultured CFs, TGF-β1 can activate STAT3 phosphorylation, increasing fibrosis-related protein expression, and relaxin can block STAT3 phosphorylation and reverse TGF-β1-induced fibrosis (Yuan et al., 2017). These results suggest that STAT3 either acts as a separate signal molecule downstream of TGF-β or interacts with the TGF-β/Smad pathway to regulate cardiac fibrosis (Figure 7).

### 4.3 MicroRNAs (miRs)

MiRs are a class of endogenous noncoding single-stranded RNAs that are about 19–25 nucleotides long. First, within the nucleus, RNA polymerase II transcribes the gene encoding the miR into the primary transcript (pri-miR). Then, the pri-miR is transported to the cytoplasm under the cooperative action of the Ran-GTP enzyme and transporter Exportin5, and the double-stranded RNA-specific nuclease Dicer enzyme cleaves the pri-miR, which is transported to the cytoplasm to form double-stranded miR of 21–25 nucleotides. The helicase unwinds the double-stranded miR, leading to degradation of one strand and the formation of a mature miR with a hydroxyl group at the 3′-end and a phosphate group at the 5′-end. Finally, the RNA-induced gene silencing complex binds the mature miR, thereby regulating target gene silencing post-transcriptionally (Lu and Rothenberg, 2018). In recent years, the relationship between miRs and pathological fibrosis has been examined, but the specific mechanisms by which miRs regulate fibrosis are still worth exploring. During the development of liver fibrosis induced by viral hepatitis, the levels of miR-16, miR-146a, miR-221, and miR-222 were markedly increased in the serum of patients with chronic hepatitis C (Abdel-Al et al., 2018). In the livers of mice treated with CCl_4_, miR-30c and miR-193 were specifically downregulated (Roy et al., 2015). Interestingly, other studies indicated that miR-29 could promote apoptosis in cardiomyocytes by downregulating antiapoptotic genes such as Bcl-2, CDC42 and Tcl-1, while miR-29 could prevent fibrosis by inhibiting the release of collagen from the ECM (Pekarsky et al., 2006; Mott et al., 2007; van Rooij et al., 2008). These results indicate that different miRs may have opposite effects on fibrosis regulation, and the same miR may have significant differences in fibrosis regulation.

STAT3 and miRs have crosstalk that is crucial for maintaining cardiac function under normal and pathological conditions. This STAT3-miR crosstalk can mediate cardiac disease in several ways. First, STAT3 can directly bind to miRs to mediate a feedback regulatory relationship or mediate an indirect feedback regulatory relationship with miRs through a long noncoding RNA (lncRNA)/protein. As an example, in oxygen-glucose deprivation-induced cardiomyocyte injury, lncRNA MIAT, which is associated with myocardial infarction, captures miR-181a-5p and boosts the expression of JAK2. This, in turn, amplifies myocardial inflammation and apoptosis through the JAK2/STAT3 signaling pathway (Tan et al., 2021). In addition, miR-21 activates the STAT3 signaling by targeting tumor suppressor cell adhesion molecule 1 (CADM1) and enhances cardiac fibrosis (Cao et al., 2017). Second, STAT3 can directly mediate the transcription of downstream miRs, and phosphorylated STAT3 can cooperate with other transcription factors to promote or inhibit the transcription of miRs. In diabetic hearts exposed to ischaemia/reperfusion, STAT3 has the ability to attach to the miR-17–92 promoter and stimulate the targeted inhibition of pro-apoptotic prolyl hydroxylase 3 (PHD3) by miR-17/20a, resulting in a decrease in apoptosis (Samidurai et al., 2020). Moreover, phosphorylated STAT3 can interact with NF-κB and inhibit miR-188-3p expression (Kuo et al., 2017; Sp et al., 2018; Masoumi-Dehghi et al., 2020). Third, miRs specifically recognize the 3′UTR of STAT3 mRNA and form incomplete complementary pairing, resulting in the inhibition of STAT3 mRNA translation, thereby blocking STAT3 expression. Following myocardial infarction, the expression of STAT3 mRNA is reduced by miR-17-5p and miR-124, which leads to the deterioration of autophagy, inflammation, myocardial remodeling, and apoptosis. These miRs bind to the 3′UTR of STAT3 mRNA (He et al., 2018; Chen et al., 2022). In summary, multiple miRs can interact with STAT3 through different mechanisms to enhance or inhibit cardiac fibrosis (Figure 7).

### 4.4 Other mediators impact cardiac fibrosis through the STAT3 signaling pathway

In addition to the above mediators that can affect cardiac fibrosis through the STAT3 signaling pathway, there are other mediators that can affect myocardial fibrosis caused by ischemia/reperfusion, atrial fibrillation, diabetic heart disease, DCM, and hypertensive heart damage through the STAT3 signaling pathway (Table 2).

**TABLE 2:** Mediators regulate fibrosis through the STAT3 signaling pathway.

Mediators	Models	Effects and related mechanisms	Reference
SHP-1	SHP-1-overexpressing myocytes and fibroblasts	The use of STAT3 agonist colivelin leads to more ROS generation, ECM deposition, and TGF-β1/SMAD2 activation	Zang et al. (2023)
	Hypoxia/reoxygenation induced cardiomyocytes	Y-box protein 1 knockdown attenuates acute myocardial infarction damage via SHP-1 mediated STAT3 suppression	Cao et al. (2020)
PTEN	Coronary artery ischemia/reperfusion model in Type 1 diabetes rats induced by Streptozotocin	PTEN partially inhibits the post ischemic regulation and post hypoxic regulation of diabetes heart through destroying JAK2/STAT3 signaling pathway	Xue et al. (2016)
βIV spectrin	Cardiac specificity βIV spectrin KO mice	βIV spectrin deficiency in cardiomyocytes causes STAT3 impairment, fibrosis, and impaired cardiac function	Unudurthi et al. (2018)
	Genetic and acquired mouse models of βIV-spectrin deficiency	βIV spectrin protein dysfunction leads to nuclear STAT3 accumulation and activation, which changes gene expression and CF behavior. Fibrosis and cardiac dysfunction in βIV spectrin-deficient mice are abolished by STAT3 inhibition	Patel et al. (2019)
Elabela	Ang II induced myocardial hypertrophy and fibrosis exacerbation in hypertensive mice	By inhibiting the IL-6/STAT3/GPX4 signaling pathway, antagonize the promoting effects of Ang II mediated cardiac microvascular endothelial cells deionization, adverse myocardial remodeling, fibrosis, and cardiac dysfunction	Zhang et al. (2022b)
PPAR	Type 1 diabetes rat model induced by Streptozotocin	PPARδ activation might suppress STAT3 and lower connective tissue growth factor and Fibronectin levels in diabetic rats with cardiac fibrosis	Lo et al. (2017b)
	PPARα knockout mice	PPARα blocks T helper 17 cell differentiation via IL-6/STAT3/RORγT pathway, thus alleviating autoimmune Myocarditis	Chang et al. (2019)
SIRT3	SIRT3 knockout mice	SIRT3 can inhibit the STAT3-NFATc2 signaling pathway, thereby reducing myofibroblast transdifferentiation and preventing cardiac fibrosis	Guo et al. (2017)

**Abbreviation:** SHP-1, tyrosine phosphatase 1; ECM, extracellular matrix; ROS, active oxygen; TGF-β1, transforming growth factor-β1; SMAD2, small mother against decapentaplegic 2; PTEN, phosphatase and tensin homologue deleted on chromosome 10; CF, cardiac fibroblasts; GPX4, glutathione peroxidase; PPAR, peroxisome proliferator-activated receptor.

## 5 The regulatory role of STAT3 and autophagy in cardiac fibrosis

Autophagy is widely present in eukaryotic organisms and is a process that degrades harmful substances in cells and promotes their recycling through the lysosome pathway. In general, moderate autophagy can maintain the stability of the internal environment, while excessive autophagy can induce cell damage (Kuma et al., 2017). The process is mainly divided into four stages: induction, initiation, elongation, and mature degradation, which are regulated by complex molecular mechanisms (Estrada-Navarrete et al., 2016; Liu et al., 2016; Lin et al., 2019; Kaushal et al., 2020). Autophagy recovers and removes damaged proteins and organelles, playing an important role in maintaining the normal function of myocardial cells (Mialet-Perez and Vindis, 2017). Interestingly, the role of autophagy in fibrosis may vary with fibrosis progression. Zhang et al. found that inhibiting autophagy could improve myocardial fibrosis in mice subjected to TAC surgery (Zhang et al., 2021). At 20 weeks after TAC in mice with endothelial leptin receptor gene knockout, myocardial fibrosis in these mice was improved by autophagy activation (Gogiraju et al., 2019). These research results demonstrate that the activation or inhibition of autophagy may occur during the process of cardiac fibrosis, and the role of autophagy in fibrosis has a dual nature.

Autophagy could potentially be linked to numerous signaling pathways, one of which is the STAT3 signaling pathway that governs the fate of cells, determining whether they survive or perish. Yuan et al.'s research indicates that relaxin attenuates TGF-β1-induced autophagy in primary CFs by suppressing the phosphorylation of STAT3, thereby reducing cardiac fibrosis (Yuan et al., 2017). In septic cardiomyopathy, the reduced expression of miR-125b leads to excessive activation of STAT3/high mobility group box protein 1 (HMGB1), resulting in elevated ROS generation and impaired autophagic flow, ultimately leading to myocardial dysfunction (Yu et al., 2021). Additionally, the overexpression of Src-associated in mitosis 68 (Sam68) promotes the osteogenic differentiation of human valvular interstitial cells (hVICs) through the STAT3 signaling-mediated autophagy inhibition, thus inducing aortic valve calcification, while knockdown of Sam68 reduces the phosphorylation of TNF-α-activated STAT3 and the expression of downstream genes, thereby affecting autophagic flow in hVICs (Liu et al., 2023b). The activation of STAT3 is crucial for reducing cardiac autophagy and inhibiting cardiac ischemia/reperfusion injury, as demonstrated by the inhibition of soluble receptor for advanced glycation end-products on cardiac ischemia/reperfusion injury (Dang et al., 2019).

## 6 Challenges and opportunities for targeting the STAT3 signaling pathway for the treatment of fibrosis

Targeting STAT3 for heart disease treatment presents significant challenges. STAT3 is widely recognized for its role in promoting myocardial fibrosis. However, myocardial fibrosis may not always be detrimental in certain heart diseases. Excessive fibrosis, for instance, can lead to adverse remodeling in myocardial infarction patients, potentially resulting in heart failure. Yet, in the early stages of myocardial infarction, fibrosis is crucial in maintaining the structural integrity of the infarcted ventricle (Prabhu and Frangogiannis, 2016). Moreover, STAT3 actively participates in the activation and proliferation of CFs, fostering fibrotic remodeling. In cardiomyocytes, STAT3 exhibits a dual nature. It can offer protective or adverse effects, such as enhancing survival and mitigating oxidative stress or mediating cardiac hypertrophy (Wang et al., 2021; Li et al., 2022). Despite cardiomyocytes not being directly involved in ECM production, they can influence the fibrotic response through paracrine signals (Qu et al., 2017). Additionally, the STAT3 signaling pathway interacts with other pathways, playing varying roles. JAK1, for example, binds to TGF-βR1, while JAKs also associate with gp130 and get activated by TGF-β (Itoh et al., 2018). Previous studies have shown that STAT3 works in tandem with Smad3 to induce connective tissue growth factor, contributing to fibrosis (Liu et al., 2013; Tang et al., 2017). Conversely, overactivated STAT3 signaling in lung fibroblasts diminishes SMAD signaling by reducing Smad3 phosphorylation, potentially due to Smad7 induction, although this theory requires experimental validation (O'Donoghue et al., 2012). Thus, identifying the optimal timing for STAT3 inhibition is crucial for maximizing therapeutic benefits and minimizing side effects. Targeting STAT3 in CFs could effectively reduce fibrosis, but its protective potential in cardiomyocytes warrants consideration. Overall, STAT3’s role in cardiac biology is multifaceted. A thorough understanding of its function across various cell types and disease stages is essential for developing effective treatments.

Despite the complexities in targeting STAT3 signaling for fibrosis treatment, recent advancements have yielded promising results (Table 3). Presently, methods to directly inhibit STAT3, aimed at targeting fibrosis, are categorized based on various target domains. These include the SH2, DBD, NTD, and TAD. In this section, we highlight key STAT3 inhibitors that specifically target these domains of the STAT3 protein.

**TABLE 3:** STAT3 inhibitors for treating organ fibrosis.

Classification	Inhibitor name	Target site	Mode of targeting STAT3	Fibrotic organs treated	Reference
Small molecules	Stattic	SH2	Phosphorylation	myocardium, liver, lung, kidney	Celada et al. (2018), Dong et al. (2019), Fu et al. (2019), Park et al. (2022)
	S3I-201	SH2	Dimerization	myocardium, lung, liver	Chen et al. (2017b), Wang et al. (2018), Yuan et al. (2023)
	BP-1–102	SH2	Dimerization	kidney	Zhu et al. (2019)
	STX-0119	NTD	DNA binding	liver, kidney	Choi et al. (2019), Makitani et al. (2020)
	Niclosamide	Unknown	Unknown	liver, lung, kidney	Chen et al. (2021), Cui et al. (2021), Gan et al. (2023)
Natural compounds	Cucurbitacin I	SH2	Phosphorylation	liver	Hu et al. (2020a)
	Cryptotanshinone	SH2	Phosphorylation	myocardium, liver, lung	Lo et al. (2017a), Zhang et al. (2019a), Zhao et al. (2022)

### 6.1 Inhibitors targeting the SH2 domain

STAT3 homodimerization is facilitated by protein-protein interactions between the SH2 domains of the individual monomers, particularly via phosphorylation at Tyr705. This pivotal molecular interaction has been harnessed to develop inhibitors targeting STAT3 directly (Furtek et al., 2016). Inhibiting the SH2 domain not only disrupts STAT3 activation and dimerization but also impedes its subsequent nuclear translocation and the expression of genes regulated by STAT3.

Several small molecule STAT3 inhibitors, notably Stattic, S3I-201, and S3I-201 analogs, play a significant role in mitigating myocardial fibrosis. These inhibitors function by binding to the SH2 domain of STAT3, thereby curtailing its activity. Elevated levels of fibroblast growth factor 23 (FGF23) are reported to induce atrial fibrosis in atrial fibrillation patients through enhancing ROS production and subsequent STAT3 and Smad3 phosphorylation. Stattic has been shown to counteract these effects (Dong et al., 2019). Moreover, administering S3I-201 to mice with myocardial infarction has demonstrated reduced left atrial fibrosis *in vivo* (Chen et al., 2017b).

Another category of inhibitors targeting STAT3’s SH2 domain comprises derivatives of natural compounds. Cryptotanshinone, a primary active component extracted from Salvia miltiorrhiza, suppresses the STAT3 pathway to reduce cardiac fibrosis and improve cardiac function in diabetic rats (Lo et al., 2017a). *In vitro* studies reveal that cryptotanshinone significantly curbs Ang II-induced cardiomyocyte hypertrophy and TGF-β-induced myofibroblast activation by impeding STAT3 phosphorylation and nuclear translocation (Li et al., 2023). Additionally, natural compounds like curcumin and resveratrol have been identified to possess properties beneficial in combating atherosclerosis (Zordoky et al., 2015; Ganjali et al., 2017).

These inhibitors are crucial for their anti-inflammatory and anti-atherosclerotic properties, suggesting their potential as therapeutic agents for ameliorating fibrosis. However, these inhibitors are not without drawbacks. A primary issue is that most inhibitors targeting the SH2 domain lack specificity to STAT3, making it challenging to exclude the involvement of other STAT proteins in fibrosis (Szelag et al., 2016). Additionally, STAT3 monomers or unphosphorylated STAT3 proteins can interact with other proteins to transcribe downstream target genes, which limits the efficacy of targeting the SH2 domain. Further complicating matters, activating mutations in the SH domain have been identified in somatic cells. The impact of these somatic mutations on the binding efficiency of SH2 domain inhibitors to STAT3, and consequently on their effectiveness, remains to be fully understood (Qiu and Fan, 2016). Therefore, the precise targeting of STAT3’s SH2 domain warrants further research focus.

### 6.2 Inhibitors targeting the DBD domain

The DBD of STAT3 specifically recognizes and binds to distinct DNA elements in target genes. This selective interaction facilitates the precise induction of target gene expression, characterized by high specificity.

Research has uncovered that platinum compounds, including IS3-295, CPA-1, CPA-7, and platinum tetrachloride (IV), effectively block the DNA-binding activity of STAT3. These compounds can inhibit cell growth and induce apoptosis, while not affecting normal cells and avoiding prolonged STAT3 activation (Beebe et al., 2018). Additionally, Galiellalactone, a natural product, impedes STAT3’s DNA-binding activity by interacting with its DBD domain. To enhance its oral bioavailability, N-acetyl L-cysteine methyl ester has been added to the thiol group, resulting in the creation of the prodrug GPA512. However, GPA512’s lack of specificity, as it also disrupts other signaling pathways like NF-κB and TGF-β, could pose challenges in its future development (Don-Doncow et al., 2014; Escobar et al., 2016). InS3-54, discovered through an advanced computer screening method, selectively binds to STAT3’s DBD domain *in vitro*, inhibiting its DNA-binding activity. Its analog, InS3-54A18, exhibits improved solubility, specificity, and pharmacological properties, while showing minimal side effects in animal models (Huang et al., 2016).

While virtual screening techniques, including molecular modeling, have demonstrated that certain inhibitors can directly bind to the DBD domain of STAT3, the scarcity of adequate assay systems has limited the identification of small molecule inhibitors in this category. This constraint has significantly impeded the drug development process. Additionally, inhibitors targeting the STAT3 DBD encounter similar challenges to those faced by SH2 domain-targeting inhibitors in terms of therapeutic application.

### 6.3 Inhibitors targeting NTD and TAD domains

Inhibitors targeting the NTDs and TAD of STAT3 can modulate the binding of STAT3 dimers and regulate DNA transcription, potentially contributing to anti-fibrotic effects. In the study of the selective STAT3 NTD inhibitor ST3-H2A2, Timofeeva et al. observed that this compound robustly activated apoptosis genes, leading to the induction of apoptosis in cancer cells (Timofeeva et al., 2013). Moreover, researchers have successfully identified the allosterically active small molecule K116, which binds to the TAD of STAT3 and effectively inhibits its activity (Huang et al., 2018).

In summary, while numerous STAT3 inhibitors have demonstrated anti-fibrotic properties, identifying inhibitors that are highly efficient, low in toxicity, and have minimal side effects remains a challenge. Additionally, there is a scarcity of extensive animal studies on the pharmacology and toxicology of these inhibitors. Furthermore, only a limited number of these inhibitors have progressed to clinical evaluation. However, the integration of STAT3 inhibitors with other targeted therapeutic agents, particularly in combination with immunotherapy agents, offers promising potential. It is hoped that future research will lead to significant advancements, enabling the broader clinical application of STAT3 inhibitors.

## 7 Conclusion

Cardiac fibrosis results from the excessive accumulation of ECM in the myocardium and is central to many cardiac pathologies. Since JAK/STAT3 activation can increase fibrotic effector cells and ECM deposition through various pathways, it may be a potential target of antifibrotic therapy. As mentioned previously, we emphasized the promoting effects of various mediators on cardiac fibrosis through activation of the JAK/STAT3 signaling pathway. However, there may be many other mediators that have not yet been identified, and modern proteomics technology and protein identification will speed up the discovery. Regarding fibrosis, the antifibrotic effect of STAT3 inhibitors is receiving attention, but there has been little research on their ability to inhibit myocardial fibrosis. While further research is required to elucidate its role in various types of myocardial fibrosis, the JAK/STAT3 signaling holds promise as a therapeutic target for cardiac fibrosis due to its connection between cardiac inflammation and fibrosis.
